# Accuracy of Direct Antimicrobial Susceptibility Testing of Gram-Negative Bacteria from Positive Blood Cultures Using MicroScan System and Value of Using Expert Rules for β-Lactam Agents

**DOI:** 10.1128/aac.02148-21

**Published:** 2022-03-15

**Authors:** Michael R. Jacobs, Caryn E. Good, Ayman M. Abdelhamed, Robert A. Bonomo

**Affiliations:** a Department of Pathology, Case Western Reserve University, Cleveland, Ohio, USA; b Department of Pathology, University Hospitals Cleveland Medical Center, Cleveland, Ohio, USA; c Department of Medicine, Case Western Reserve University, Cleveland, Ohio, USA; d Department of Molecular Biology and Microbiology, Case Western Reserve University, Cleveland, Ohio, USA; e Department of Pharmacology, Case Western Reserve University, Cleveland, Ohio, USA; f Department of Biochemistry, Case Western Reserve University, Cleveland, Ohio, USA; g Center for Proteomics and Bioinformatics, Case Western Reserve University, Cleveland, Ohio, USA; h Louis Stokes Cleveland Department of Veterans Affairs Medical Center, Cleveland, Ohio, USA; i Case Western Reserve University-Cleveland Veterans Affairs Medical Center for Antimicrobial Resistance and Epidemiology, Cleveland, Ohio, USA

**Keywords:** ESBL, carbapenemase, direct antimicrobial susceptibility, expert rules

## Abstract

Direct antimicrobial susceptibility testing (AST) of positive blood cultures with Gram-negative bacteria produces results within 24 h, compared to 48 to 96 h with conventional methods. Positive clinical blood cultures were studied, supplemented with contrived blood cultures inoculated with a spectrum of resistant isolates. Bacterial inocula used for direct AST were quantitated. Direct AST was performed using MicroScan NM43 trays inoculated directly from positive blood cultures (100 μL in 25 mL water) and incubated using a WalkAway instrument, with trays read after 16 h. Reference AST was performed the following day from growth on solid medium using the same trays. Agreement of AST results between direct and reference methods, with and without the use of three expert rules for β-lactams, was evaluated using FDA categorical agreement criteria. Of 86 specimens tested (41 clinical specimens and 45 contrived specimens), the mean bacterial load in positive blood cultures was 8.98 log_10_ CFU/mL. Fifteen isolates contained extended-spectrum β-lactamases, and 27 contained carbapenemases. Of 1,985 pairs of AST categorical results for 25 antimicrobials, 55.0% were susceptible, 4.7% intermediate, and 40.4% resistant by reference testing. Overall categorical agreement was 92.3%, with 5.3% minor errors, 1.9% major errors, and 0.4% very major errors. Agreement was higher for non-β-lactam agents (95.8%) than for β-lactam agents (90.3%; *P *< 0.0001). Application of expert rules increased agreement for β-lactam agents to 94.6%. The methods used achieved the study goal of producing accurate, cost-effective AST results directly from positive blood cultures using MicroScan trays with a 16-h incubation time without the need for additional testing. Use of three expert β-lactam rules improved accuracy.

## INTRODUCTION

Detection of bacteremia by blood culture is currently considered the gold standard for the diagnosis of bloodstream infections (BSIs), with early determination of phenotypic resistance patterns being essential for patient management strategies (1). The evolution of β-lactamases, particularly extended-spectrum β-lactamases (ESBLs) and carbapenemases, has produced clinical challenges in sepsis and limited the coverage of β-lactams such as piperacillin-tazobactam and cefepime, which are commonly used for empirical therapy (2, 3). A recent study documented correlations between the prevalence of resistance for different combinations of antibiotics and bacteria and hospitalization and mortality rates in adults (4). Examples of Gram-negative bacterial species showing these correlations included species of *Enterobacterales* that were resistant to expanded-spectrum cephalosporins, carbapenems, and fluoroquinolones. Determination of the antimicrobial susceptibility profile of isolates is important for management of patients with BSIs, providing information needed for escalation or deescalation of antimicrobial therapy based on the results. With conventional methods, antimicrobial susceptibility testing (AST) results are available 48 to 96 h after blood cultures become positive, while rapid methods can provide results within 6 to 24 h (5).

A wide variety of rapid methods are now available for identification of pathogens and AST or detection of genes associated with resistance directly from positive blood cultures (2, 6, 7). Rapid methods for bacterial identification include matrix-assisted laser desorption ionization–time of flight mass spectrometry (MALDI-TOF MS) and PCR arrays, while rapid methods for AST include PCR arrays and bacterial-growth-based phenotypic assays (8). PCR arrays currently available have limited bacterial and resistance gene targets, while MALDI-TOF MS can identify a broad spectrum of bacterial species. Rapid phenotypic AST methods also have limited antimicrobial targets, and significant issues with rapid methods are their cost and technical complexity. Few rapid methods eliminate the need for conventional AST to confirm and supplement their findings.

One aim of our study was to perform bacterial identification and AST of positive blood cultures in a cost-effective and rapid manner for common Gram-negative pathogens without the need for conventional AST to supplement the findings. These goals were implemented by using a gene array method for detection of common Gram-negative pathogens followed by use of a commercial AST panel of 25 agents incubated for 16 h, with a total turnaround time of <24 h. While this protocol is not as fast as other methods, it offers a relatively simple and cost-effective solution to providing complete AST profiles for the most frequent bloodstream pathogens, including species most often associated with resistance to expanded-spectrum cephalosporins and carbapenems; this method has been reported to provide rapid phenotypical profiles of antibiotic resistance and to reduce the time required to obtain traditional results (9). Another aim was to assess the need for and value of applying three expert rules to appropriate β-lactam agents for isolates producing broad-spectrum β-lactamases. These rules address derepressed AmpC, ESBL, and carbapenemase production in various genera of *Enterobacterales* (Table 1). Both of these major aims were addressed using clinical blood cultures as well as contrived blood cultures; the latter were enriched with ESBL- and carbapenemase-producing strains.

**TABLE 1:** Rules used to modify interpretative categories

Rule name	Applicable species	Rule details
Derepressed AmpC^*a*^	Enterobacter species, *Citrobacter* species,^*b*^ Klebsiella aerogenes, *Serratia* species	Change any susceptible or intermediate third-generation cephalosporins to resistant
ESBL^*c*^	Klebsiella pneumoniae, Klebsiella oxytoca, Escherichia coli, Proteus mirabilis	Change any susceptible or intermediate penicillins, cephalosporins, or aztreonam to resistant if ESBL phenotype or genotype
Carbapenemase^*d*^	All *Enterobacterales* species	Change any susceptible or intermediate carbapenems to resistant if carbapenemase-producing genotype

a CLSI M100-S31, Table 2A, comment 17, page 37 (16).

b This rule was not applied to Citrobacter koseri.

c CLSI M100-S31, Table 3A, comment 17, reporting, page 115 (16).

d CLSI M100-S31, Table 3B-1, reporting, page 124 (16).

## RESULTS

A total of 86 unique isolates were tested, 41 from clinical specimens and 45 from contrived specimens. The bacterial species of these isolates and their ESBLs and carbapenemases are shown in Table 2. Fifteen isolates of *Enterobacterales* contained ESBLs (11 CTX-M, 2 SHV-based, and 2 TEM-based) and 22 contained carbapenemases (18 KPC and 4 NDM). Four strains of Acinetobacter baumannii contained OXA carbapenemase, and 1 strain of Pseudomonas aeruginosa contained VIM carbapenemase.

**TABLE 2:** Bacterial species found and presence of ESBLs and carbapenemases in study isolates

Genus/species	No. of isolates from clinical/contrived specimens	No. with β-lactamase type:						
		CTX-M	SHV ESBL	TEM ESBL	KPC	NDM	OXA	VIM
*Citrobacter* ^*a*^	3/3	–^*b*^	–	–	3	–	–	–
Enterobacter */* K. aerogenes ^*c*^	1/4	–	–	–	2	2	–	–
Escherichia coli	20/17	7	1	2	6	2	–	–
Klebsiella pneumoniae	12/9	4	1	–	7	–	–	–
Proteus mirabilis	3/0	–	–	–	–	–	–	–
Acinetobacter baumannii	1/9	–	–	–	–	–	4	–
Pseudomonas aeruginosa ^*d*^	1/3	–	–	–	–	–	–	1
Total	41/45	11	2	2	18	4	4	1

a C. freundii (*n* = 3) and C. koseri (*n* = 3).

b –, none.

c E. cloacae (*n* = 4) and K. aerogenes (*n* = 1).

d Two isolates were carbapenem resistant, associated with OMP deletions.

**FIG 1:**
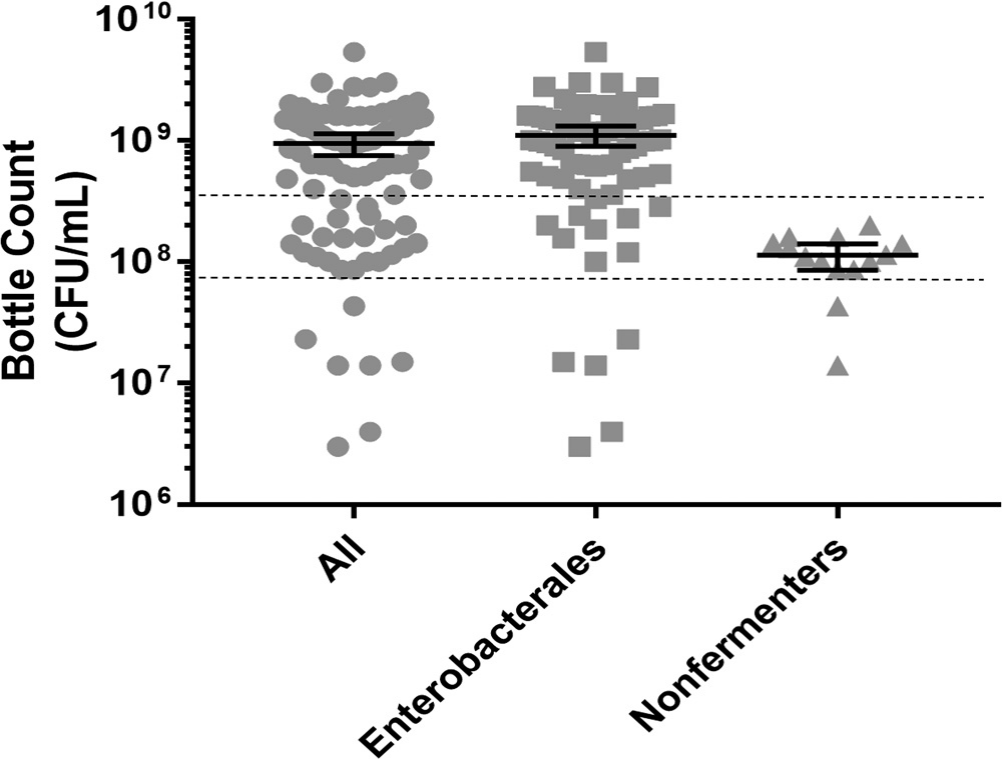
Bacterial loads present in positive blood culture bottles. Bars indicate mean values and 95% CIs. Dashed lines indicate the target inoculum range for 0.5 McFarland standard bacterial suspensions, diluted 1:250 (100 μL added to 25 mL of Pluronic water), to produce inocula of 3 × 10^5^ to 5 × 10^5^ CFU/mL.

Inocula present in the positive blood culture bottles at the time of direct AST ranged from 6.3 to 9.54 log_10_ CFU/mL (mean, 8.98 log_10_ CFU/mL [95% confidence interval [CI], 8.84 to 9.12 log_10_ CFU/mL]) (Fig. 1). The mean inoculum density was significantly higher for *Enterobacterales* (9.05 log_10_ CFU/mL [95% CI, 8.91 to 9.18 log_10_ CFU/mL]), compared to nonfermenters (8.05 log_10_ CFU/mL [95% CI, 7.99 to 8.12 log_10_ CFU/mL]; *P *< 0.05).

**FIG 2:**
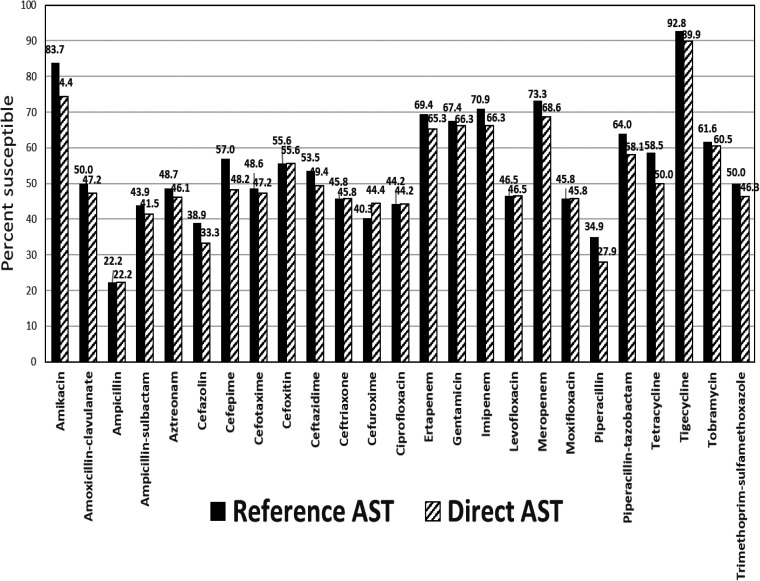
Susceptibility of isolates to antimicrobial agents by reference AST and direct AST.

Overall, 1,985 pairs of AST categorical results were valid based on the bacterial species tested, with 55.0%, 4.7%, and 40.4% of isolates being susceptible, intermediate, and resistant, respectively, by reference testing and 52.7%, 4.2%, and 44.0% by direct testing (see Table S2 in the supplemental material). The susceptibility of isolates by reference and direct AST for each antimicrobial agent tested is shown in Fig. 2. Except for cefuroxime, all agents had higher susceptibility rates by reference testing. Differences in resistance rates between methods were highest for amikacin, amoxicillin-clavulanate, cefepime, cefoxitin, imipenem, meropenem, and piperacillin-tazobactam (see Table S2). There were 4 *Enterobacterales* isolates with cefepime susceptible-dose dependent (SDD) interpretations; all produced either a carbapenemase (1 Citrobacter freundii isolate) or an ESBL (2 Escherichia coli isolates and 1 Klebsiella pneumoniae isolate).

Categorical agreement and error rates of testing by reference and direct AST methods are shown in Table 3. The overall categorical agreement was 92.3%, with 5.3% minor errors (mEs), 1.9% major errors (MEs), and 0.4% very major errors (VMEs). Categorical agreement was higher for non-β-lactam agents than for β-lactam agents (95.8% versus 90.3%; *P *< 0.0001). Application of expert rules for β-lactam agents increased the agreement of β-lactams significantly (90.3% versus 94.6%; *P* < 0.0001), with the categorical agreement rate being comparable to that of non-β-lactam agents (94.6% versus 95.8%; *P *= 0.27).

**TABLE 3:** Categorical agreement of direct and reference AST results for non-β-lactams and β-lactams, with and without application of expert rules

Antimicrobial agent group	No. of result pairs	No. (%) of pairs with:			
		Categorical agreement	mEs	MEs	VMEs
Non-β-lactam agents	735	704 (95.8)	21 (2.9)	7 (1.0)	3 (0.4)
β-Lactam agents	1,250	1,129 (90.3)	85 (6.8)	31 (2.5)	5 (0.4)
β-Lactam agents with expert rules	1,250	1,182 (94.6)	58 (4.7)	9 (0.7)	1 (0.1)
All agents^*a*^	1,985	1,833 (92.3)	106 (5.3)	38 (1.9)	8 (0.4)

a Based on β-lactam agents without expert rules. For non-β-lactam agents versus β-lactam agents, *P* < 0.0001, *P* = 0.0002, and *P* = 0.03 for categorical agreement, mEs, and MEs, respectively; for β-lactam agents with versus without expert rules, *P* < 0.0001, *P* = 0.03, and *P* = 0.0008 for categorical agreement, mEs, and MEs, respectively; for non-β-lactam agents with expert rules versus β-lactam agents with expert rules, *P* = 0.27, *P* = 0.7, and *P* = 0.8 for categorical agreement, mEs, and MEs, respectively.

Categorical agreement and error rates for individual antimicrobial agents are shown for non-β-lactams in Table S3 and for β-lactams in Table S4. Most errors for non-β-lactams were minor (21/31 errors), with all MEs occurring with amikacin (*n* = 4) and trimethoprim-sulfamethoxazole (*n* = 3). Most errors for β-lactams were also minor (85/121 errors), with agreement being significantly improved by the use of expert rules. The largest number of errors occurred with ampicillin-sulbactam (10 mEs) and cefoxitin (11 mEs).

All 15 *Enterobacterales* isolates with ESBL-associated genotypes were detected by the phenotypic ESBL tests used, with no off-scale results and all but 1 isolate showing phenotypic ESBL production with both cefotaxime-clavulanate and ceftazidime-clavulanate (see Table S5). Phenotypic ESBL production was also shown for 13 of 22 carbapenem-producing *Enterobacterales* isolates but was not found in any of the other isolates. Application of expert rules improved categorical agreement (94.6% versus 90.3%; *P *= 0.27), with the greatest differences being noted with cefepime, ceftazidime, and imipenem (see Table S4). Differences between categorical errors for *Enterobacterales* with and without the use of expert rules according to resistance mechanism were significantly different for isolates producing carbapenemases (*P *= 0.0006) and ESBLs (*P *= 0.0024) (Table 4). No categorical errors were found when the derepressed AmpC rule was applied to AmpC-producing species. Differences using expert rules associated with advanced-generation cephalosporins and aztreonam, as well as carbapenems, were analyzed in more detail (Table 5). This showed a significant difference between methods for advanced-generation cephalosporins and aztreonam with the carbapenemase group (*P *= 0.01) and a difference approaching significance for carbapenems with this group (*P *= 0.08). Three ESBL-producing isolates (1 E. coli isolate and 2 K. pneumoniae isolates) were intermediate to ertapenem (MICs of 1 μg/mL) but susceptible to imipenem and meropenem by direct testing and were susceptible to all three carbapenems by reference testing.

**TABLE 4:** Categorical errors for *Enterobacterales* strains with and without expert rules according to resistance mechanism

Resistance mechanism	No. of result pairs	Categorical errors without rules (%)	Categorical errors with rules (%)	*P*
Carbapenemase	352	12.5	4.8	0.0006
ESBL	240	13.8	6.7	0.0024
Other^*a*^	557	6.6	5.9	0.3
All	1,149	9.9	5.7	0.004

a Isolates without carbapenemases or ESBLs.

**TABLE 5:** Susceptibility of *Enterobacterales* strains with and without ESBLs and carbapenemases to advanced-generation cephalosporins and aztreonam and to carbapenems

Group	No. of isolates	% of isolates susceptible to:			
		Advanced-generation cephalosporins^*a*^ and aztreonam		Carbapenems^*b*^	
		Reference method	Direct method	Reference method	Direct method
ESBL^*c*^	15	6.7	1.3^*d*^	100	93.3^*e*^
Carbapenemase^*f*^	22	11.8	1.8^*g*^	15.2	4.6^*h*^
Other^*i*^	31	100	98.1	100	100

a Cefepime, cefotaxime, ceftazidime, and ceftriaxone.

b Ertapenem, meropenem, and imipenem.

c *Enterobacterales* strains with ESBLs.

d *P *= 0.1 versus reference method.

e Three ESBL-producing isolates (1 E. coli isolate and 2 K. pneumoniae isolates) were intermediate to ertapenem (MICs of 1 μg/mL). *P = *0.1 versus reference method.

f *Enterobacterales* strains with carbapenemases.

g *P = *0.01 versus reference method.

h *P = *0.08 versus reference method.

i E. coli, K. pneumoniae, and P. mirabilis strains without ESBLs or carbapenemases.

Essential agreement of the methods is shown in Table S6. This analysis was limited, because only 111 of 1,985 result pairs had on scale MIC values determined by both methods. MIC values were the same for 79 isolates (71.2%), 1 doubling dilution higher by the reference method for 20 isolates (18.0%), 2 doubling dilutions higher by the reference method for 4 isolates (3.6%), 1 doubling dilution higher by the direct method for 6 isolates (5.4%), and 2 doubling dilutions higher by the direct method for 2 isolates (1.8%). Overall essential agreement was 94.6% (105/110 result pairs). By individual agent, piperacillin-tazobactam (3/4 isolates [75.0%]) and ceftazidime (17/21 isolates [81.0%]) had the lowest rates of essential agreement.

Based on FDA criteria for agreement of AST methods, overall rates of essential (94.6%) and categorical (92.3%) agreement met the criteria for these performance determinations (>89.9%). The ME rate of 1.9% and the VME rate of 0.4% also met FDA criteria for these determinations (ME rate of <3% and VME rate of <2.4%). Categorical agreement and error rates were further improved by application of expert rules.

## DISCUSSION

The 86 isolates present in the clinical and contrived blood cultures were representative of the bacterial species most frequently recovered from patients with BSIs and included *Citrobacter* species, Enterobacter species, E. coli, K. pneumoniae, Proteus mirabilis, A. baumannii, and P. aeruginosa (Table 2). ESBL- and carbapenemase-producing isolates were well represented, with 15 isolates, all *Enterobacterales*, in the former group (11 isolates with CTX-M, 2 isolates with SHV, and 2 isolates with TEM) and 27 in the latter (18 *Enterobacterales* isolates with KPC, 4 *Enterobacterales* isolates with NDM, 4 A. baumannii isolates with OXA, and 1 P. aeruginosa isolate with VIM). AST results were generated for 1,985 organism-agent pairs, with 55.0%, 4.7%, and 40.4% of isolates being susceptible, intermediate, and resistant, respectively, to the 25 agents by reference AST, providing a good representation of resistant isolates to evaluate error rates.

Bacterial loads determined at the time of AST to assess the effect of the inoculum size used for direct AST showed that the inoculum was in the appropriate range for A. baumannii and P. aeruginosa but was 1 log_10_ unit higher for *Enterobacterales* (Fig. 1). Assessment of categorical error rates between direct and reference AST results showed 92.3% categorical agreement, with 5.3% mEs, 1.9% MEs, and 0.4% VMEs, meeting the FDA parameters for these rates (>89.9% categorical agreement with <3% MEs and <2.4% VMEs). Categorical agreement was higher for non-β-lactam agents (95.8% versus 90.3%; *P* < 0.0001) (Table 3). Lower rates of susceptibility to advanced-generation cephalosporins and carbapenems were found for both the ESBL- and carbapenemase-producing groups, compared to other *Enterobacterales* isolates (Table 4). However, 3 of 22 ESBL-producing isolates were intermediate to ertapenem by direct testing.

Application of the three expert rules used increased categorical agreement for β-lactam agents from 90.3% to 94.6% (*P *< 0.0001), comparable to that for non-β-lactam agents (95.8%; *P *= 0.27) (Table 3). Of the 152 result pairs with categorical errors, 106 had mEs, 38 MEs, and 8 VMEs. Error rates showed the same patterns found with categorical agreement, with the use of expert rules decreasing the number of errors from 121 to 68, with only 1 VME (with cefoxitin) for the β-lactam group with the use of expert rules. The use of expert rules considerably improved agreement between the methods for aztreonam, cefepime, ceftazidime, imipenem, and meropenem (see Table S4 in the supplemental material). The ESBL rule used covers the most common ESBL-producing species but does not address ESBL production in other species, which is increasing, and attention to this issue is needed.

Many studies and guidance documents support the use of the three expert rules used in our study. The derepressed AmpC rule is supported by recommendations to avoid expanded-spectrum cephalosporins for infections caused by *Enterobacterales* strains with inducible AmpC β-lactamases (10, 11). A recent review of evidence for the efficacy of various β-lactams in the treatment of BSIs caused by ESBL-producing *Enterobacterales* strains concluded that carbapenems should be used, rather than β-lactam-β-lactamase inhibitor combinations or cephalosporins (12). A study of BSIs caused by ceftriaxone-resistant E. coli and K. pneumoniae strains showed a higher mortality rate with piperacillin-tazobactam than with meropenem, providing evidence for extending the ESBL rule to include β-lactam-β-lactamase inhibitor combinations (13). Recent Infectious Diseases Society of America guidance for treatment of ESBL-producing *Enterobacterales* and carbapenem-resistant *Enterobacterales* (CRE) strains also provides support for the use of the ESBL and CRE rules (14). This guidance document recommends carbapenems for treatment of BSIs due to ESBL-producing *Enterobacterales* strains, noting that piperacillin-tazobactam and cefepime should be avoided, even if *in vitro* susceptibility is demonstrated. Carbapenems are not recommended for carbapenemase-producing *Enterobacterales* strains in BSIs, although extended-infusion meropenem is recommended for meropenem-susceptible, ertapenem-resistant isolates that do not produce carbapenemases.

Use of a gene array method for identification of bacterial species present in positive blood cultures and detection of resistance genes in clinical specimens was used in our study but is not essential and can be replaced by other methods to achieve the same objectives. For identification, MALDI-TOF MS can be used directly on early growth of subcultures from positive blood cultures (15). Phenotypic detection of ESBL production was very accurate and has the advantage of detecting TEM- and SHV-based ESBLs that are not readily detected by current gene array systems (8). Phenotypic carbapenemase detection based on interpretation of carbapenem MICs was also accurate, with only 3 false-positive carbapenem results, all with ertapenem; this can be resolved by using one of the many rapid carbapenemase detection methods available as needed (8, 16, 17).

From these observations, we concluded that the large inoculum used for direct testing of *Enterobacterales* did not adversely affect AST results and that the three expert rules significantly improved the accuracy of testing. This improvement with the use of expert rules was likely associated with better expression of β-lactam resistance in the direct testing group, with higher rates of resistance to advanced-generation cephalosporins and carbapenems (Table 4). The β-lactams with the greatest differences between the direct and reference methods were amoxicillin-clavulanate, cefepime, cefoxitin, imipenem, meropenem, and piperacillin-tazobactam (see Table S2). Livermore et al. stated that, despite assertions by CLSI and EUCAST, with low breakpoints susceptibility results for cephalosporins and carbapenems can be reported “as found,” even for strains with ESBLs and carbapenemases, and they noted that “it is prudent to continue to seek ESBLs and carbapenemases directly and, where they are found, generally to avoid substrate drugs as therapy” (18). Our findings strongly support this statement.

Studies of other methods of direct AST have shown similar agreement for the agents tested, but most of those studies tested far fewer resistant isolates and considerably fewer antimicrobial agents. A study published in 1998 evaluated direct AST of 114 Gram-negative isolates using MicroScan Combo 15 panels inoculated with bacterial suspensions obtained by centrifugation of positive blood cultures (19). In that study, 16.6% of 2,250 organism-agent combinations were resistant, with categorical agreement of 94.7%. In a recent study evaluating the Accelerate Pheno system and direct VITEK2 testing, with next-day VITEK2 testing being used as the reference method, 131 specimens were tested against 11 agents, with categorical agreement of 93.4% and 97.4%, respectively. However, only 18.6% of the 1,191 organism-drug results were resistant by the reference method (compared to 40.4% in our study), and 87.8% of errors with both rapid methods (158/180 errors) occurred with the seven β-lactam agents tested (20). A recent study of 40 positive blood cultures evaluated a MicroScan tray similar to the one used in our study by direct inoculation, with next-day testing using the same tray as the reference method (9). That study evaluated reading trays at 4, 6, and 8 h, as well as the standard time of 16 h, and found 60%, 65%, 67.5%, and 100% agreement at those times, respectively, concluding that 16 h of incubation is optimal and noting that detection of resistance, but not susceptibility, at earlier times was valid and could be valuable.

Overall, our study showed that direct AST from positive blood cultures could be successfully used, with categorical agreement and error rates comparable to those of reference testing as well as other rapid, direct methods, meeting FDA guidelines for performance of AST systems. The strengths of our study include the large number of antimicrobial agents tested, the large proportion of resistant organism-agent tests, with inclusion of typical resistance mechanisms now encountered, such as ESBLs and carbapenemases, and the use of expert rules, which was shown to improve results. Limitations of our study include that it was performed in a single institution in the United States, that essential agreement assessment was limited by the range of antimicrobial concentrations present in the trays used, and that resistance mechanisms common in other regions were not studied.

In conclusion, the methods used achieved the study goals of producing accurate, cost-effective AST results directly from positive blood cultures using MicroScan trays with a 16-h incubation time without the need for additional testing of commonly used antimicrobial agents. Application of three expert rules for β-lactam agents further improved the accuracy of the AST results.

## MATERIALS AND METHODS

### Clinical blood cultures.

Blood cultures collected using BacT/Alert FA Plus (aerobic) or SN (anaerobic) bottles (bioMérieux, Inc., Durham, NC) were incubated using the BacT/Alert VIRTUO system (bioMérieux). Positive, deidentified, clinical blood cultures with Gram-negative bacilli seen with Gram staining were tested using Verigene BC-GN arrays (Luminex, Austin, TX) using software version 2.6.0b1. Bottles with Acinetobacter species, *Citrobacter* species, Enterobacter species, Escherichia coli, Klebsiella pneumoniae, Klebsiella oxytoca, Proteus species, or Pseudomonas aeruginosa detected were entered into the study, with a limit of one bottle per patient. Definitive identification of bacterial isolates was performed from growth on subculture by MALDI-TOF MS (Bruker Daltonics GmbH & Co., Billerica, MA) using MALDI Biotyper CA version 3.2 build 14 software. University Hospitals Cleveland Medical Center Institutional review board approval for the study was obtained.

### Contrived blood cultures.

Selected characterized, stored isolates of A. baumannii, *Citrobacter* species, Enterobacter species, E. coli, K. pneumoniae, K. oxytoca, and P. aeruginosa were recovered from frozen storage and used to inoculate BacT/Alert FA Plus or SN bottles. Isolates were chosen to supplement clinical specimens with less-common species and strains with ESBLs and carbapenemases. Bottles were inoculated with 0.1 mL of organism suspensions (diluted to contain 10 to 100 CFU/mL) and 10 mL of reconstituted human blood (4 mL of packed red blood cells and 6 mL of plasma). Inoculated bottles were incubated using the VIRTUO system (bioMérieux) as for clinical specimens.

### Direct AST.

Direct AST was performed within 6 h of bottles being flagged as positive by the VIRTUO system (bioMérieux). AST was performed using MicroScan NM43 trays (Beckman Coulter, Brea, CA), which included 25 agents relevant to blood isolates (see Table S1 in the supplemental material). Trays also contained cefotaxime-clavulanate (0.5/4, 4/4, and 8/4 μg/mL) and ceftazidime-clavulanate (0.25/4 and 2/4 μg/mL), with isolates showing a ≥3-fold concentration decrease in the MIC of either cephalosporin alone interpreted as positive for ESBL production. Trays were inoculated with bacterial suspensions prepared using 100 μL from positive blood culture bottles added to 25 mL of stabilized aqueous Pluronic suspending solution (Beckman Coulter) (1:250 dilution). Trays were incubated and read after incubation for 16 h using the MicroScan WalkAway system (Beckman Coulter) using LabPro version 4.42 panel update 7 software. Bacterial inocula present in blood culture bottles at the time of AST were determined by serial dilution and plating of bottle contents according to standard methods.

### Reference AST.

Blood culture bottles were subcultured onto blood agar plates and incubated aerobically at 35°C overnight. Isolated colonies were suspended in saline to a 0.5 McFarland standard determined spectrophotometrically, and AST was performed using MicroScan NM43 trays (Beckman Coulter) inoculated with 100 μL of each suspension in 25 mL of suspending solution. Trays were incubated and read as above.

### Expert rules.

Three expert rules, addressing AmpC-producing species and ESBL- and carbapenemase-producing *Enterobacterales*, were applied to applicable β-lactam results as shown in Table 1, based on Clinical and Laboratory Standards Institute (CLSI) procedures (16).

### Data analysis.

Blood culture bottle inocula and AST MIC and categorical results by direct and reference methods were entered into Excel worksheets. MICs were interpreted according to CLSI M100-S30 breakpoints except for cefotaxime, ciprofloxacin, and levofloxacin, for which susceptible breakpoints were adjusted to 1 dilution higher, with no intermediate values, based on the concentration ranges of these agents in the panel used, and tigecycline, for which FDA breakpoints were used (see Table S1) (16, 21). Categorical and essential agreement rates were calculated according to FDA criteria, with categorical errors between reference and direct methods being characterized as mEs (intermediate result by one method and either susceptible or resistant by the other method), MEs (false resistant result), or VMEs (false susceptible result) (22). Categorical errors were determined for data with and without the use of expert rules. Essential agreement was defined as agreement (within ±1 dilution [2-fold]) of direct and reference MICs for all result pairs for which both values are on scale. The performance of direct AST was assessed based on FDA criteria, as follows: (i) essential and categorical agreement rates should be >89.9%; (ii) the ME rate, based on the number of susceptible organisms tested, should be <3%; and (iii) the VME rate, based on the number of resistant organisms tested, should be <2.4% (22). The significance of differences between reference and direct AST results was determined by Pearson’s chi-squared test with Yates correction for continuity or Fisher’s exact test, with *P* values of ≤0.05 being regarded as significant.
