# Supplementary material for: Accuracy of Direct Antimicrobial Susceptibility Testing of Gram-Negative Bacteria from Positive Blood Cultures Using MicroScan System and Value of Using Expert Rules for β-Lactam Agents
Source: Antimicrob Agents Chemother. 2022 Mar 15;66(3):e02148-21. doi: 10.1128/aac.02148-21 (PMC8923179; doi:10.1128/aac.02148-21)
Supplement: Supplemental file 1 — Supplemental tables. Download aac.02148-21-s0001.pdf, PDF file, 0.3 MB [file aac.02148-21-s0001.pdf]

Table S1. Antimicrobial agents tested and interpretative breakpoints

		Interpretative breakpoints S/I/R[1] (μg/mL)		
**Antimicrobial agent**	**Concentration range tested (μg/mL)**	**Enterobacterales**	**Acinetobacter baumannii complex**	**Pseudomonas aeruginosa**
**Amikacin**	**8-32**	**≤16/32/>32**	**≤16/32/>32**	**≤16/32/>32**
**Amoxicillin-clavulanate[2]**	**8-16**	**≤8/16/>16**	**-[3]**	**-**
**Ampicillin**	**2-16**	**≤8/16/>16**	**-**	**-**
**Ampicillin-sulbactam[2]**	**4-16**	**≤8/16/>16**	**≤8/16/>16**	**-**
**Aztreonam**	**4-16**	**≤4/8/>8**	**-**	**≤8/16/>16**
**Cefazolin**	**2-16**	**≤2/4/>4**	**-**	**-**
**Cefepime**	**2-16**	**≤2/4-8[4]/>8**	**≤8/16/>16**	**≤8/16/>16**
**Cefotaxime**	**2-32**	**≤2[5]/-/>4**	**-**	**-**
**Cefoxitin**	**4-16**	**≤8/16/>16**	**-**	**-**
**Ceftazidime**	**1-16**	**≤4/8/>8**	**≤8/16/>16**	**≤8/16/>16**
**Ceftriaxone**	**1-32**	**≤1/2/>2**	**-**	**-**
**Cefuroxime**	**4-16**	**≤8/16/>16**	**-**	**-**
**Ciprofloxacin**	**0.5-2**	**≤0.5[5]/-/>1**	**≤1/2/>2**	**≤0.5/1/>1**
**Ertapenem**	**0.5-4**	**≤0.5/1/>1**	**-**	**-**
**Gentamicin**	**1-8**	**≤4/8/>8**	**≤4/8/>8**	**≤4/8/>8**
**Imipenem**	**1-8**	**≤1/2/>2**	**≤2/4/>4**	**≤2/4/>4**
**Levofloxacin**	**1-4**	**≤1[5]/-/>1**	**≤1/2/>2**	**≤2/4/>4**
**Meropenem**	**1-8**	**≤1/2/>2**	**≤2/4/>4**	**≤2/4/>4**
**Moxifloxacin**	**2-4**	**≤2/4/>4**	**-**	**-**
**Piperacillin**	**16-64**	**≤16/32-64/>64**	**≤16/32-64/>64**	**≤16/32-64/>64**
**Piperacillin-tazobactam[6]**	**8-64**	**≤16/32-64/>64**	**≤16/32-64/>64**	**≤16/32-64/>64**
**Tetracycline**	**2-8**	**≤4/8/>8**	**≤4/8/>8**	**-**
**Tigecycline**	**1-4**	**≤2/4/>4**	**≤2/4/>4**	**-**
**Tobramycin**	**2-8**	**≤4/8/>8**	**≤4/8/>8**	**≤4/8/>8**
**Trimethoprim-sulfamethoxazole[7]**	**0.5-2**	**≤2/-/>2**	**≤2/-/>2**	**-**

[1] S, susceptible; I, intermediate; R, resistant
[2] Tested at 2:1 ratios
[3] -, not applicable
[4] Susceptible, dose-dependent
[5] Susceptible range adjusted based on lowest concentration present in panel, with no intermediate value, as susceptible CLSI breakpoints are now one doubling dilution lower
[6] Tested with constant tazobactam concentration of 4 μg/mL
[7] Trimethoprim component of 1:19 ratio of trimethoprim to sulfamethoxazole

Table S2. Categorical susceptibility of testing by reference and direct AST, with differences between the two methods.

		AST category by reference method (%)			AST category by direct method (%)			Difference (%)[1]		
**Antimicrobial agent**	**n**	**S**	**I**	**R**	**S**	**I**	**R**	**S**	**I**	**R**
**Amikacin**	86	83.7	5.8	10.5	74.4	8.1	17.4	9.3	-2.3	-7
**Amoxicillin-clavulanate**	72	50	11.1	38.9	47.2	5.6	47.2	2.8	5.6	-8.3
**Ampicillin**	72	22.2	4.2	73.6	22.2	2.8	75.0	0.0	1.4	-1.4
**Ampicillin-sulbactam**	82	43.9	12.2	43.9	41.5	9.8	48.8	2.4	2.4	-4.9
**Aztreonam**	76	48.7	1.3	50	46.1	1.3	52.6	2.6	0.0	-2.6
**Cefazolin**	72	38.9	2.8	58.3	33.3	4.2	62.5	5.6	-1.4	-4.2
**Cefepime**	86	57	7.0	36	48.2	1.2	50.6	8.7	5.8	-15.0
**Cefotaxime**	72	48.6	0	51.4	47.2	0	52.8	1.4	0	-1.4
**Cefoxitin**	72	55.6	16.7	27.8	55.6	9.7	34.7	0.0	6.9	-6.9
**Ceftazidime**	86	53.5	4.7	41.9	49.4	7.1	43.5	4.1	-2.4	-1.7
**Ceftriaxone**	72	45.8	0	54.2	45.8	1.4	52.8	0	-1.4	1.4
**Cefuroxime**	72	40.3	4.2	55.6	44.4	2.8	52.8	-4.2	1.4	2.8
**Ciprofloxacin**	86	44.2	1.2	54.7	44.2	1.2	54.7	0.0	0.0	0
**Ertapenem**	72	69.4	4.2	26.4	65.3	4.2	30.6	4.2	0.0	-4.2
**Gentamicin**	86	67.4	2.3	30.2	66.3	3.5	30.2	1.2	-1.2	0
**Imipenem**	86	70.9	7.0	22.1	66.3	1.2	32.6	4.7	5.8	-10.0
**Levofloxacin**	86	46.5	1.2	52.3	46.5	1.2	52.3	0.0	0.0	0
**Meropenem**	86	73.3	3.5	23.3	68.6	1.2	30.2	4.7	2.3	-7
**Moxifloxacin**	72	45.8	0.0	54.2	45.8	0	54.2	0.0	0.0	0
**Piperacillin**	86	34.9	4.7	60.5	27.9	9.3	62.8	7.0	-4.7	-2.3
**Piperacillin-tazobactam**	86	64	8.1	27.9	58.1	4.7	37.2	5.8	3.5	-9.3
**Tetracycline**	82	58.5	7.3	34.1	50.0	13.4	36.6	8.5	-6.1	-2.4
**Tigecycline**	69	92.8	5.8	1.5	89.9	7.3	2.9	2.9	-1.4	-1.4
**Tobramycin**	86	61.6	2.3	36	60.5	4.7	34.9	1.2	-2.3	1.2
**Trimethoprim-sulfamethoxazole**	82	50	0.0	50	46.3	0	53.7	3.7	0.0	-3.7
**All agents**	**1985**	**55.0**	**4.7**	**40.4**	**52.7**	**4.2**	**44.0**	**2.3**	**0.5**	**-3.6**

S, susceptible; I, intermediate; R, resistant

[1] Difference between reference and direct methods

Table S3. Categorical agreement of direct and reference AST for non-β-lactams

Antimicrobial agent	Number of result pairs	CA	mE	ME	VME
**Amikacin**	**86**	**76**	**6**	**4**	**0**
**Ciprofloxacin**	**86**	**84**	**0**	**0**	**2**
**Gentamicin**	**86**	**84**	**1**	**0**	**1**
**Levofloxacin**	**86**	**86**	**0**	**0**	**0**
**Moxifloxacin**	**72**	**72**	**0**	**0**	**0**
**Tetracycline**	**82**	**73**	**9**	**0**	**0**
**Tigecycline**	**69**	**66**	**3**	**0**	**0**
**Tobramycin**	**86**	**84**	**2**	**0**	**0**
**Trimethoprim-sulfamethoxazole**	**82**	**79**	**0**	**3**	**0**
**All non-β-lactam agents**	**735**	**632 (95.8%)**	**21 (2.9%)**	**7 (1.0%)**	**3 (0.4%)**
**CA, categorical agreement; mE, minor error; ME, major error; VME, very major error**					

Table S4. Categorical agreement of direct and reference AST for β-lactams with and without expert rules

		Categorical agreement without rules				Categorical agreement with rules				
**Antimicrobial agent**	**Number of result pairs**	**CA**	**mE**	**ME**	**VME**	**CA**	**mE**	**ME**	**VME**	**CA difference**
**Amoxicillin-clavulanate**	72	65	6	1	0	65	6	1	0	0
**Ampicillin**	72	68	3	1	0	68	3	1	0	0
**Ampicillin-sulbactam**	82	72	10	0	0	72	10	0	0	0
**Aztreonam**	76	70	2	3	1	76	0	0	0	**6 (7.9%)**
**Cefazolin**	72	67	3	2	0	68	3	1	0	1 (1.4%)
**Cefepime**	86	73	7	6	0	84	2	0	0	**11 (12.8%)**
**Cefotaxime**	72	69	0	2	1	72	0	0	0	**3 (4.2%)**
**Cefoxitin**	72	59	11	1	1	61	9	1	1	2 (2.8%)
**Ceftazidime**	86	78	4	3	1	86	0	0	0	**8 (9.3%)**
**Ceftriaxone**	72	70	1	0	1	72	0	0	0	**2 (2.8%)**
**Cefuroxime**	72	67	5	0	0	69	3	0	0	**2 (2.8%)**
**Ertapenem**	72	66	6	0	0	69	3	0	0	**3 (4.2%)**
**Imipenem**	86	76	7	3	0	85	1	0	0	**11 (12.8%)**
**Meropenem**	86	80	2	4	0	86	0	0	0	**6 (7.0%)**
**Piperacillin**	86	75	9	2	0	75	9	2	0	0
**Piperacillin-tazobactam**	86	74	9	3	0	74	9	3	0	0
**All β-lactam agents**	**1250**	**1129 (90.3%)**	**85 (6.8%)**	**31 (2.5%)**	**5 (0.4%)**	**1182 (94.6%)[1]**	**58 (4.7%)**	**9 (0.7%)**	**1 (0.1%)**	**53 (4.3%)**

CA, categorical agreement; mE, minor error; ME, major error; VME, very major error
[1] *P*<0.001 vs without rules

Table S5. Phenotypic ESBL testing of Enterobacterales

		ESBL detected with cefotaxime-clavulanate		ESBL detected with ceftazidime-clavulanate	
**Resistance mechanism**	**N**	**Reference**	**Direct**	**Reference**	**Direct**
**Carbapenemase**	22	13	**12**	**13**	**13**
**ESBL**	15	15	**15**	**14**	**15**
**Other**[1]	35	0	0	0	0
**All**	72	25	27	**27**	**28**

[1] Isolates without carbapenemases or ESBLs

Table S6. Essential agreement (EA) of AST by reference and direct methods

Antimicrobial agent	Number of result pairs	EA	EA%
**Amikacin**	**5**	**5**	100
**Amoxicillin-clavulanate**	**3**	**3**	100
**Ampicillin**	**7**	**7**	100
**Ampicillin-sulbactam**	**11**	**11**	100
**Aztreonam**	**4**	**4**	100
**Cefazolin**	**3**	**3**	100
**Cefepime**	**1**	**1**	100
**Cefotaxime**	**1**	**0**	0
**Cefoxitin**	**6**	**6**	100
**Ceftazidime**	**21**	**17**	81.0
**Ceftriaxone**	**1**	**1**	100
**Cefuroxime**	**4**	**4**	100
**Ciprofloxacin**	**3**	**3**	100
**Ertapenem**	**0**	**0**	-
**Gentamicin**	**11**	**11**	100
**Imipenem**	**0**	**0**	-
**Levofloxacin**	**3**	**3**	100
**Meropenem**	**1**	**1**	100
**Piperacillin**	**2**	**2**	100
**Piperacillin-tazobactam**	**4**	**3**	75.0
**Tetracycline**	**10**	**10**	100
**Tigecycline**	**6**	**6**	100
**Tobramycin**	**3**	**3**	100
**Trimethoprim-sulfamethoxazole**	**1**	**1**	100
**Total**	**111**	**105**	94.6
